# Extended π-conjugative n-p type homostructural graphitic carbon nitride for photodegradation and charge-storage applications

**DOI:** 10.1038/s41598-019-43312-5

**Published:** 2019-05-10

**Authors:** Devthade Vidyasagar, Sachin G. Ghugal, Suresh S. Umare, Murali Banavoth

**Affiliations:** 1 Materials and Catalysis Laboratory, Department of Chemistry, Visvesvaraya National Institute of Technology (VNIT), Nagpur, 400010 India; 2 Solar Cells and Photonics Research laboratory, School of Chemistry, University of Hyderabad, Hyderabad, Telangana 500046 India

**Keywords:** Photocatalysis, Photocatalysis

## Abstract

An n-p type homostructural metal-free graphitic carbon nitride (g-C_3_N_4_) semiconductor is designed and developed for pollutant abatement and energy storage application. The successful grafting of vibrio-like morphology-based g-C_3_N_4_ by 2, 5-Thiophenedicarboxylic acid (TDA) molecule and the development of amide-type linkage substantiated the prosperous uniting of g-C_3_N_4_ with organic TDA moiety is demonstrated. An extended π-conjugative TDA grafted g-C_3_N_4_ exhibited band gap tunability with broadband optical absorbance in the visible region. Mott-Schottky analysis exhibited the formation of n-p type homostructural property. As a result, obtained TDA grafted g-C_3_N_4_ has extended π-conjugation, high surface area and adequate separation of charge carriers. The change in the photocatalytic performance of grafted g-C_3_N_4_ is inspected for degradation of acid violet 7 (AV 7) dye under visible light irradiation. The charge storage capacity of grafted g-C_3_N_4_ was additionally assessed for supercapacitive behaviour. The charge capacitive studies of grafted g-C_3_N_4_ exhibited the areal capacitance of 163.17 mF cm^−2^ and robust cyclic stability of 1000 cycles with capacity retention of 83%.

## Introduction

Graphitic carbon nitride (g-C_3_N_4_) as analogous to graphene, has semiconductor characteristics with a band gap of ~2.7 eV, owing to sp^2^ hybridised carbon and nitrogen atoms arranged in a six-member stalked rings^1,2^. Due to excellent thermal and chemical stability with unique optoelectronic properties, g-C_3_N_4_ predominantly studied for photocatalytic water splitting^3–7^, oxidative degradation of air/water pollutants^8,9^, bacterial inactivation^10–13^, reduction of carbon dioxide^14,15^, organic synthesis^16,17^ and as well as in electrochemical devices^18,19^. As an alternative to metallic photocatalyst, g-C_3_N_4_ expedited interest as non-toxic and facilely available inexpensive material for a broad variety of photo/electro-catalytic applications^2,20^. There are extensive reports on the utilisation of “g-C_3_N_4_ as photocatalyst” and are systematically summarised in sundry of inspiring reviews^21–26^. Albeit considerable progress is made in g-C_3_N_4_ chemistry, the photocatalytic performance of bulk g-C_3_N_4_ is still obstructed by poor visible light absorption, high recombination of electron-hole pairs and low surface area^26^. Therefore, several approaches have been applied to enhance the photocatalytic performance of g-C_3_N_4_. Two categories of modification were adopted to alter the structural and physicochemical properties of g-C_3_N_4_. Mainly pre and post-modification of g-C_3_N_4_ were investigated to improve its photocatalytic performance^27^. Pre-modification includes copolymerization of melon with a felicitous external additive to tune and acquire the desired property in g-C_3_N_4_^28^. In this regard, elemental doping and polymerisation with organic conjugated moieties proved to be propitious in enhancing the activity of g-C_3_N_4_^29–32^. While post-modification involves g-C_3_N_4_ heterojunctions, where bulk g-C_3_N_4_ lattice fabricated by thermal condensation method is composited with well-matched energy band of suitable semiconductor metal oxides to form a semiconductor-semiconductor hybrid heterostructure for promotion of charge carriers from g-C_3_N_4_ to another semiconductor or vice versa^33,34^. Post-functionalization offers the benefit of grafting target function groups or dopant into g-C_3_N_4_ lattice. Post-modification of g-C_3_N_4_ is a straightforward strategy to form heterojunctions with metal oxides, where nitrogen-rich acidic site of melon units coordinate to form a covalent bond between nitrogen and metals. However, low solubility and high chemicals resistivity of g-C_3_N_4_ in prevalence organic solvents is a fundamental barrier for post-functionalization of this material. On the other hand, *in-situ* co-polymerisation of organic groups with melon units induces supramolecular pre-organisation of functional moieties into the g-C_3_N_4_ lattice structure^35,36^. Pre-functionalization of melon precursors with suitable organic molecules may produce excess defect sites or incomplete g-C_3_N_4_ framework^37^. Besides, the stability of target functional group at high temperature as 550 °C is crucial for modification of g-C_3_N_4_ derivatives. Precedent reports revealed that post-functionalization of g-C_3_N_4_ polymers offer control over the stability of functional group, prevent the undesired defect site generation and preserve basic structural unit of pristine g-C_3_N_4_^38–40^.

Thus, here we utilize the post-functionalisation strategy to incorporate 2, 5-thiophene dicarboxylic acid (TDA) moiety into the defect sites of g-C_3_N_4_. A simple solvent evaporation technique was performed to condense the free –NH_2_ group of g-C_3_N_4_ with carboxylic –OH groups of TDA moiety. Tailoring of g-C_3_N_4_ optical properties with extended π-conjugation is the motif of present work. We employed pre and post-modification strategies to functionalise g-C_3_N_4_ with TDA moiety and optimized the synthetic conditions. The improvements in photocatalytic performance of TDA-functionalised g-C_3_N_4_ are demonstrated by considering degradation of Acid violet 7 (AV 7) dye. Two-dimensional g-C_3_N_4_ is made up of earth abundant composition, low cost processing, high surface area, and eco-friendly nature. Importantly it shows electrical double layer capacitive behavior suitable for supercapacitive applications. Nevertheless, the surface to volume ratio of bulk g-C_3_N_4_ is poor, producing lower charge-capacitance properties. The addition of aromatic TDA moiety to the terminal –NH_2_ groups of tri-s-triazine units in perpendicular direction protects the restacking of g-C_3_N_4_ layers and could open up high intra-layered catalytic active sites for charge-storage behavior. Therefore, the effect of extended π-conjugation in TDA grafted g-C_3_N_4_ is additionally examined by charge capacity retention studies.

## Results and Discussion

**Figure 1:**
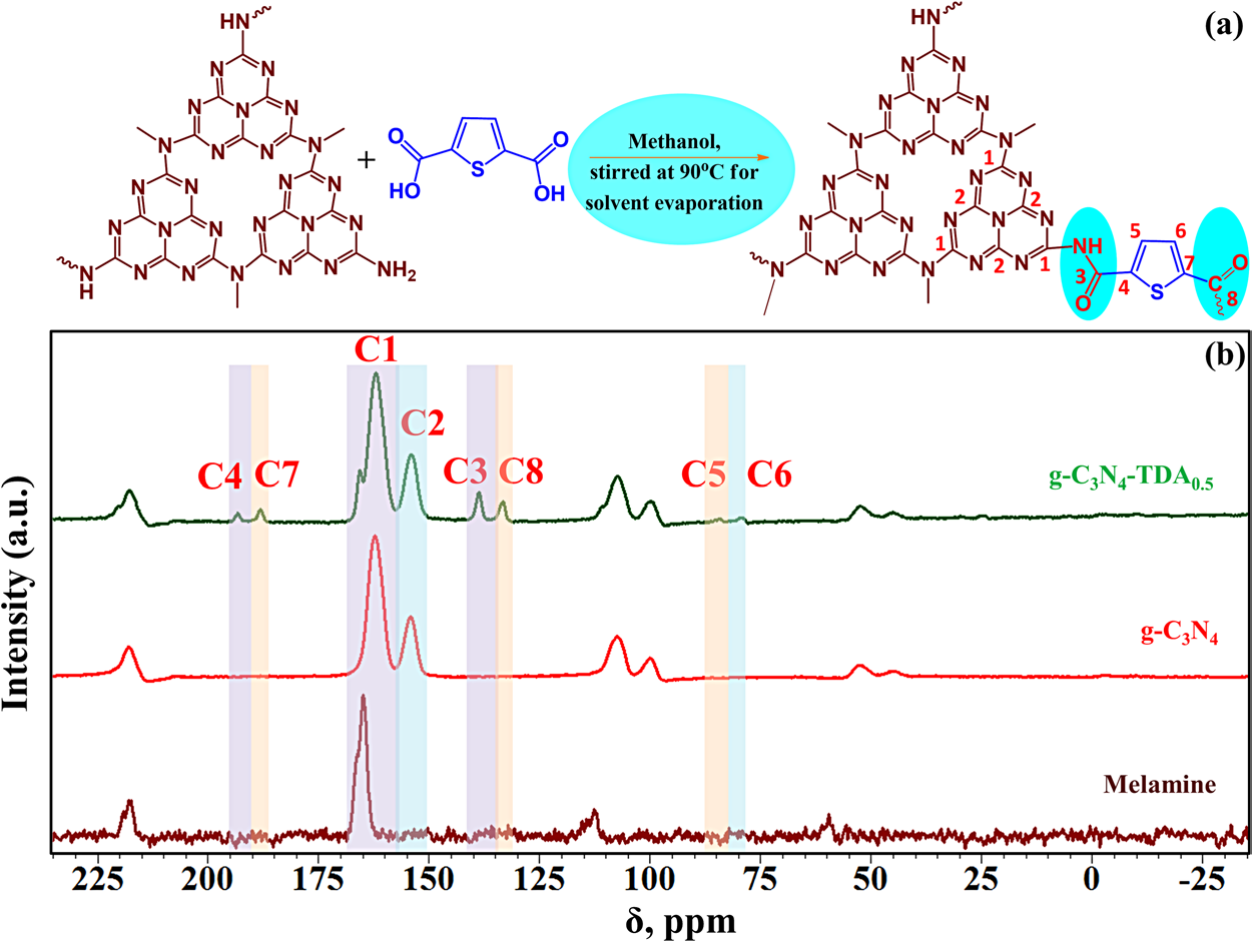
(**a**) The possible reaction involved in post-molecular grafting of g-C_3_N_4_. (**b**) Cross-polarization magic angle spinning solid-state ^13^C nuclear magnetic resonance spectra (CP-MAS ^13^C NMR) of melamine, g-C_3_N_4_ and g-C_3_N_4_-TDA_0.5_.

The condensation of g-C_3_N_4_ with TDA in methanol solvent provide the advantage of its greater methanol tolerance^41^. Concealing surface defects of g-C_3_N_4_ can improve charge separation of electron and holes with better light absorption. The surface structural modification can also alter the electronic band structure of g-C_3_N_4_ and modified band structure can promote its photocatalytic behaviour. Grafting of π-rich aromatic thiophene onto defect sites of g-C_3_N_4_ can have a pronounced effect on the electronic structure, optical properties and photocatalytic properties of the material. In principle, free –NH_2_ group of g-C_3_N_4_ and –COOH group of TDA condense to form amide type linkage as given in Fig. 1a. Polymeric g-C_3_N_4_ with terminal amino functionalities as electron donating groups endows n-type conductivity in the material. The substitution of terminal –NH/NH_2_ groups in g-C_3_N_4_ structure with electron accepting groups may induce p-type conductivity. The condensation of terminal electron donating –NH_2_ groups of g-C_3_N_4_ with an electron accepting carbonyl group might transform g-C_3_N_4_ into homostructural donor-acceptor complex with n-p type conductivity. The condensation of –NH_2_ groups ascribable to the donor and acceptor (D-A) complex formation, which brings adjacent electron deficient-electron rich rings into close proximity.

Thus, the formation of an amide type linkage could extend π-conjugation in g-C_3_N_4_. The extended conjugation of π-bonds favours the molecule to absorb visible light in the longer wavelength and TDA grafted g-C_3_N_4_ may possess n → π transition of -NH-C=O bond and π → π^*^ transition of –C=O bond. Moreover, an increase in conjugation raises the energy level of the HOMO and lowers the energy of LUMO, which in turn cause less absorption energy for an electron transition. Presence of sulfur in thiophene molecule could stimulate physicochemical properties of g-C_3_N_4_-TDA. Several sulfur-doped g-C_3_N_4_ results found that sulfur can induce a change in the electronic structure of g-C_3_N_4_ and can also contribute to the reduction in HOMO-LUMO band gap energy^42–44^. Figure. 1b is discussed in later sections.

**Figure 2:**
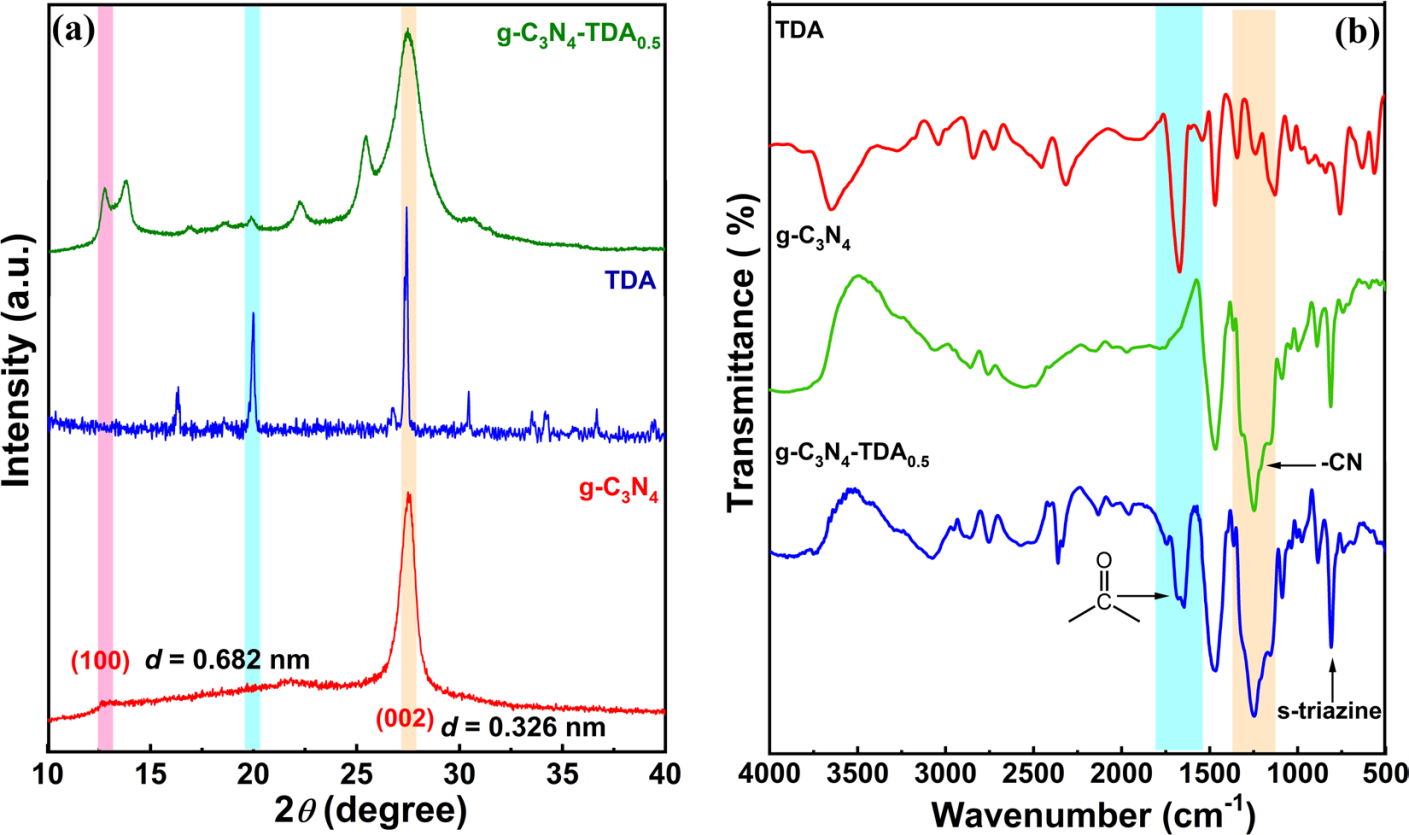
(**a**) Powder X-ray diffraction pattern and (**b**) FT-IR spectra of g-C_3_N_4_, TDA and g-C_3_N_4_-TDA_0.5_.

Powder x-ray diffraction (XRD) analysis was performed to determine the lattice structure of g-C_3_N_4_-TDA samples. The XRD patterns of g-C_3_N_4_ and TDA grafted g-C_3_N_4_ represent distinctively different diffraction patterns, a broadening of *hkl* (002) peak in g-C_3_N_4_-TDA_0.5_ indicate the absence of long-range ordering of atomic arrangements, partial collapse of intra-layer g-C_3_N_4_ units and decreased crystallinity **(**Fig. 2a**)**^16,45^. The XRD pattern of TDA has shown a sharp diffraction peak at a 2θ value of 27.2° and few other underdeveloped peaks. However, comprehension of the TDA crystal structure is still unclear. The powder XRD pattern of TDA grafted g-C_3_N_4_-TDA_0.5_ reflected several new peaks at around 2θ angle of 14.0, 23.2 and 25.1° which are likely due to the change in crystal structure caused by the grafting of organic TDA moiety. Furthermore, a broad peak at 2θ = 12.8 in g-C_3_N_4-_TDA_0.5_ sample corresponds to *hkl*(100) plane, arising from in-plane stacking of tri-s-triazine motif^46^. Another possible reason for the broadening of (002) and (100) planes is attributed to restricted stacking of g-C_3_N_4_ layers by the addition of π-electron rich TDA molecule. These results suggest the successful grafting of g-C_3_N_4_ by TDA molecule *via* direct heating in methanol solvent.

In order to verify the successful grafting of g-C_3_N_4_ with organic TDA moiety fourier transform infrared spectroscopy (FTIR) analysis were performed. In the FTIR spectra **(**Fig. 2b**)**, the –NH, –CN and s-triazine subunit stretching frequencies in g-C_3_N_4_-TDA_0.5_ appeared at ca. 3400 cm^−1^ (broad), ca. 1268 cm^−1^ (broad), and ca. 804 cm^−1^ (sharp), respectively. These characteristic stretching vibrations ensure the existence of undisturbed g-C_3_N_4_ framework in the prepared samples. Besides, the formation of a new vibration of >C=O band at ca. 1679 cm^−1^ (weak) was appeared, which clearly indicates the condensation of –COOH group and formation of terminal amide type linkage in grafted g-C_3_N_4_. Also, the absence of characteristic stretching vibrations of an acid group also supports the condensation of –COOH functional group in TDA.

Morphological alterations in TDA modified g-C_3_N_4_ was imaged using a field emission scanning electron microscope (FE-SEM). The FE-SEM image of bulk g-C_3_N_4_ depicts typical graphene sheets like aggregations (Fig. S1a). While TDA-g-C_3_N_4_ shows semi-rolled thick layer organisation, this could be due to the addition of *π*-acceptor TDA molecule; which enforced the tilting of 2D-graphene sheets (Fig. S1c). The semi-rolled thick layer structure is as a result of combined intramolecular hydrogen bonding and charge transfer interactions with TDA molecule. Furthermore, energy dispersive x-ray analysis (EDS) confirmed the elemental composition of TDA in the modified g-C_3_N_4_ (Fig. S1b). In comparison to pristine g-C_3_N_4_, TDA-g-C_3_N_4_ sample have displayed very intense carbon peak (Fig. S1d). This excess carbon weight percentage in g-C_3_N_4_ is principally due to additional TDA segment. These observed morphological changes support the grafting of g-C_3_N_4_ with organic TDA molecule.

**Figure 3:**
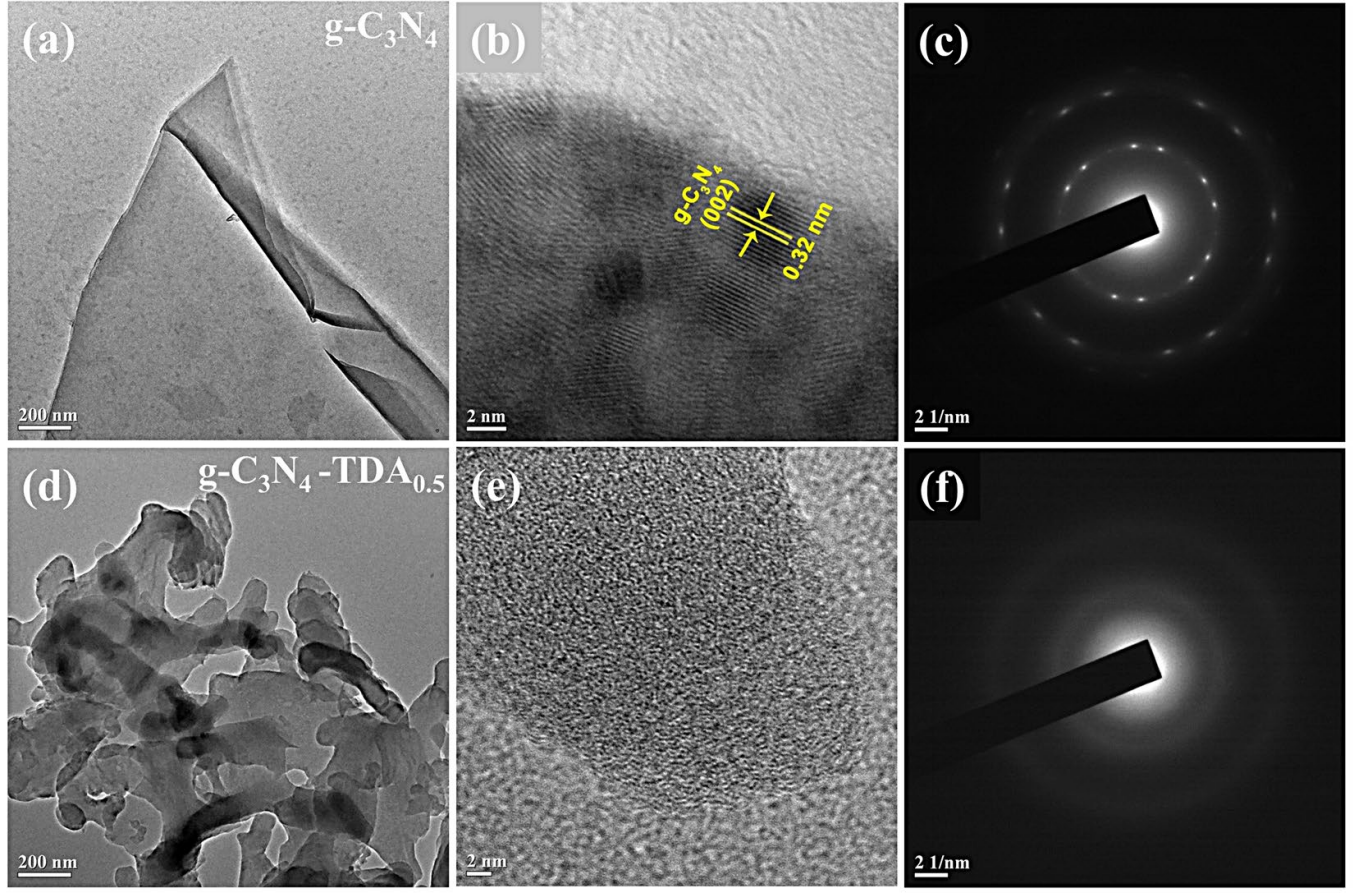
TEM image, HRTEM and SAED patterns of bulk g-C_3_N_4_ (**a**–**c**) and g-C_3_N_4_-TDA_0.5_ (**d**–**f**).

In comparison with the smooth layer structure of pristine g-C_3_N_4_, TEM image of g-C_3_N_4_-TDA_0.5_ exhibited vibrio-like branch morphology **(**Fig. 3a,d**)**. The grafting of TDA could drive to reduce the g-C_3_N_4_ terminal –NH_2_ groups and develop branch-like structures on the surface of g-C_3_N_4_ sheets, which could boost the interaction of reactants with catalytic sites. The inter-planar *d*-spacing calculated for bulk g-C_3_N_4_ was found to be 0.32 nm, and the absence of clear lattice fringes in g-C_3_N_4_-TDA_0.5_ indicates its distinctive amorphous nature **(**Fig. 3b,e**)**. Furthermore, the selected area electron diffraction (SAED) pattern of g-C_3_N_4_ reflected polycrystalline nature with a few bright spots **(**Fig. 3c**)**. These bright spots disappeared in g-C_3_N_4_-TDA_0.5,_ and it exhibited two diffuse concentric rings of maximum intensity with no discrete reflections **(**Fig. 3f**)**. This feature implies the absence of long-range order in atomic lattice of grafted g-C_3_N_4_^47^. The structural change from polycrystalline to amorphous by grafting of an organic molecule is may be due to terminal amidization which allow this change in microstructure. The surface terminal grafting of g-C_3_N_4_ with TDA molecules can act as a pillar between stalked g-C_3_N_4_ layers and prevents the self-aggregation of bulk layers. The homogeneous amorphization of g-C_3_N_4_-TDA_0.5_ is possibly due to the introduction of aromatic-TDA molecules in the perpendicular direction of the triazine network, which induces a significant decrease in the crystallinity of the material.

The chemical environment of carbon atoms in prepared samples was further investigated by solid-state cross-polarised magic angle spinning ^13^C-NMR analysis **(**Fig. 1b**)**. ^13^C NMR spectra of melamine, pristine g-C_3_N_4_ and g-C_3_N_4_-TDA of optimised compositions were recorded to study the nature of the interaction between g-C_3_N_4_ and TDA. Polymeric g-C_3_N_4_ motif is made up of continuous chains of heptazine core units where two non-equivalent sets of carbons are present. The ^13^C NMR spectrum of the pristine g-C_3_N_4_ exhibited two signals centred at 162.22 and 154.07 ppm, which can be assigned to the resonances for the CN_2_(NH_2_) and CN_3_ groups of the heptazine units^48,49^, respectively. These two peaks are in good agreement with the chemical shift of carbons in heptazine (single carbon nitride unit) molecule (164–166 ppm and 155–156 ppm)^50^. Similarly, the spectra of g-C_3_N_4_-TDA_0.5_ show the two signals at the same position as C1 (162.01 ppm) and C2 (153.88 ppm) confirming the presence of heptazine core. The peak shift appears at C4 (193.17 ppm), and C7 (187.95 ppm) can be assigned to the ketonic carbon and acidic carbon, respectively. The peak shift appearance at C5 (84.4 ppm) and C6 (79.5 ppm) can be assigned to the carbon peaks in TDA moiety. The C3 (138.63 ppm) and C8 (133.15 ppm) peak shifts can be assigned to the presence of ketonic carbon of TDA moiety in the g-C_3_N_4_-TDA_0.5_ samples. These results infer that there is a strong interaction exists between the g-C_3_N_4_ and TDA in the prepared composite. This interaction facilitates the formation of n-p type homojunction results in improving the photocatalytic performance.

**Figure 4:**
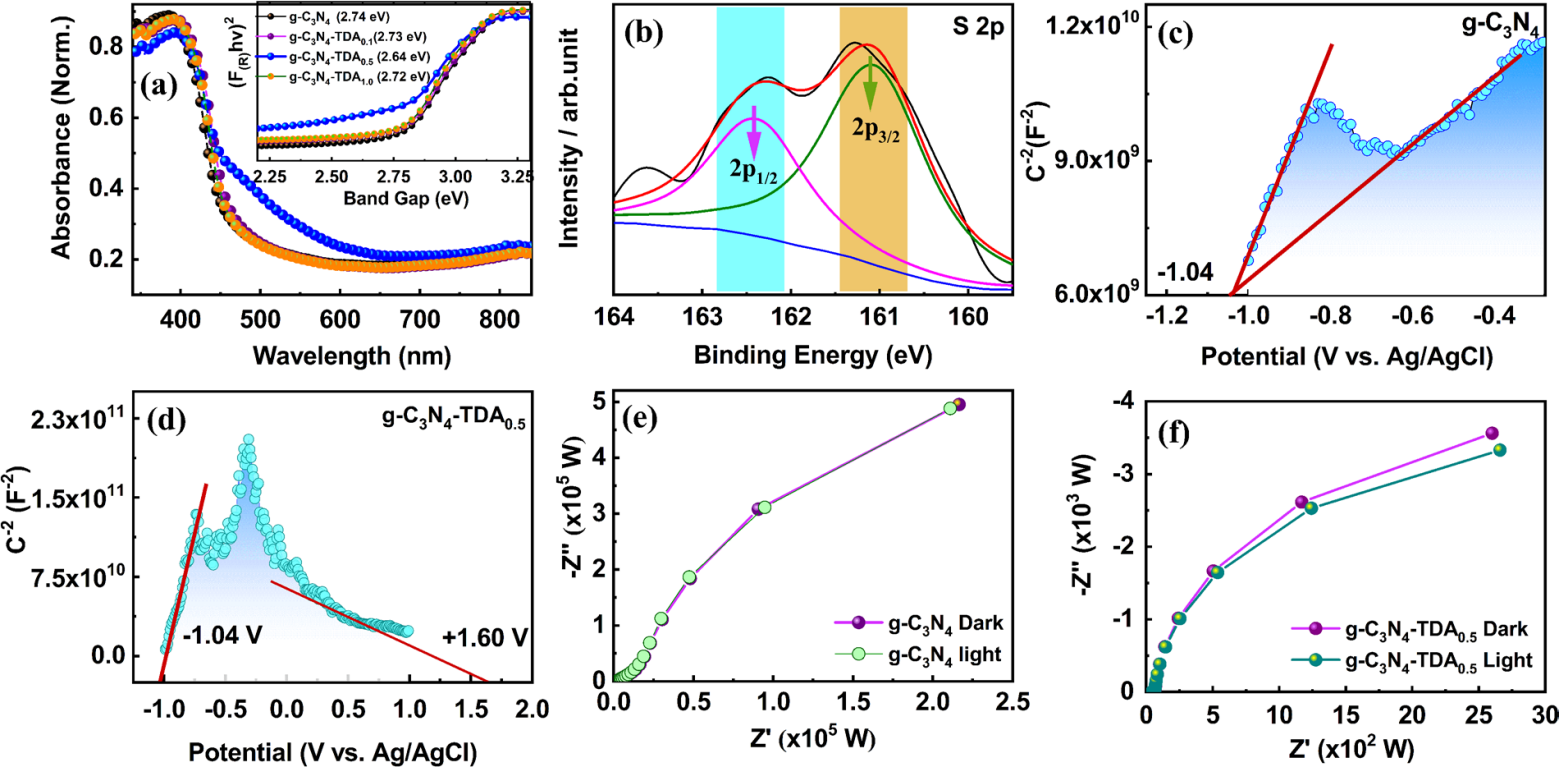
(**a**) UV-Visible absorbance, inset Kubelka-Munk plots of bulk g-C_3_N_4_ and g-C_3_N_4_-TDA_*x*_ samples (**b**) XPS profile of S2p sulfur of g-C_3_N_4_-TDA_0.5_ photocatalyst (**c**,**d**) Mott-Schottky plots and (**e**,**f**) EIS Nyquist plots of g-C_3_N_4_ and g-C_3_N_4_-TDA_0.5_ under dark and visible irradiation.

The optical properties of g-C_3_N_4_-TDA samples and bulk g-C_3_N_4_ were studied by UV-Visible diffused reflectance spectroscopy (UV-DRS). The UV-Vis spectrum g-C_3_N_4_-TDA_0.5_ sample exhibit redshift of approximately 15–30 nm than the bulk g-C_3_N_4_ (Fig. 4a). To understand the redshift of the absorbance peak, it is essential to examine the molecular structure in detail. The electronic structure of g-C_3_N_4_-TDA is comprised of electron-accepting –C=O thiophene segment, connected to the g-C_3_N_4_ unit through an amide (-NH-C=O) type linkage. The observed redshift is possibly due to an intramolecular charge-transfer mechanism (ICTM). In general, g-C_3_N_4_ acts as an n-type semiconductor with free -NH/NH_2_ groups as electron donors^51^. Addition of intramolecular *π*-acceptor TDA group to free -NH/NH_2_ terminal could effectively transfer electrons and facilitate ICTM. This resultsg-C_3_N_4_-TDA_0.5_ to act as both n-p type homojunction semiconductor. In recent years, the term “n-p homostructural g-C_3_N_4_ semiconductor” has often used to describe the charge transfer mechanism in carbon nitride systems^52^. Following this, g-C_3_N_4_-TDA_0.5_ showed a red shifted band at 490 nm, this could be as result of π → π^*^ transitions in g-C_3_N_4_ and carbonyl group of TDA acceptor segment; favouring ICTM. It is also assumed that the formation of the amide type bond extends π-conjugation and improves the light harvesting ability. To understand the change in band structure; the acquired diffused reflectance spectrum is converted to Kubelka-Munk function, the band gap of bulk g-C_3_N_4_ and g-C_3_N_4_-TDA were calculated by the transformed Kubelka-Munk plots of (F_(R_)*hv*)^1/2^ *vs*. photon energy. Considerable reduction in band gap was observed for TDA grafted g-C_3_N_4_ (Fig. 4a, inset). Further, in comparison of bulk g-C_3_N_4_ about 0.11 eV decrease in band gap was obtained for g-C_3_N_4_-TDA_0.5_ owing to a reduction in the HOMO-LUMO gap.

The surface composition and chemical state of elemental constituents in g-C_3_N_4_-TDA_0.5_ were observed by x-ray photoelectron spectroscopy (XPS). On the survey spectrum of g-C_3_N_4_-TDA_0.5_, four elements (C, N, O and S) were observed (Fig. S2). The high-resolution XPS in the S2p region of the g-C_3_N_4_-TDA_0.5_ spectrum is fitted as a single doublet (Fig. 4b). In accordance with the spin-orbit splitting effect, thiophene bound sulfur is mainly composed of 2p_3/2_ and 2p_1/2_ peaks in 2:1 intensity ratio^53^. Thus, thiophene bound to g-C_3_N_4_ has main strong binding peaks at 162.4 (2p_1/2_) and 161.0 eV (2p_3/2_). The similar binding peaks were observed for reported thiophene samples on gold^54^ or aromatic sulfur-containing self-assembled monolayers^55^. Moreover, the absence of surface sulfur binding energy peaks at 164.0 eV^53^, clearly express the existence of thiophene bound sulfur in the g-C_3_N_4_-TDA_0.5_ sample.

The Mott–Schottky (M-S) analysis of g-C_3_N_4_-TDA_0.5_ sample revealed n-p type conductive property. The M-S plot of bulk g-C_3_N_4_ exhibited a positive slope which is a typical feature of n-type semiconducting material and the flat band potential determined to be −1.04 V vs Ag/AgCl (Fig. 4c). In contrast, the M-S plot of g-C_3_N_4_-TDA_0.5_ represents a straight line with slopes in both positive and negative regimes (Fig. 4d). This could be a result of effective donor-acceptor (D-A) densities at the interface between TDA and g-C_3_N_4_ in the grafted g-C_3_N_4_-TDA_0.5_ sample. This result suggests the synchrony of n-p type conductive behaviour in g-C_3_N_4_-TDA_0.5_. Moreover, the coexistence of n- and p-type domains can benefit in effective separation and opposite migration of charged electron-hole pairs, thereby high photocatalytic performance.

To probe the charge-transfer resistance behaviour in grafted g-C_3_N_4_ samples, the electrochemical impendence (EIS) analysis was conducted in the dark and under visible light irradiation. The EIS Nyquist plot of g-C_3_N_4_-TDA_0.5_ exhibited a small radius arc than bulk g-C_3_N_4_, indicating improved electronic conductivity; lower charge transfer resistance and adequate separation of photogenerated electron-hole pairs (Fig. 4e,f). The EIS plot g-C_3_N_4_-TDA_0.5_ under visible light irradiation reflects much smaller semicircular arc than pristine g-C_3_N_4_. These observations suggest the enhanced interfacial charge transfer and effective separation of charge carriers over the surface of the grafted g-C_3_N_4_-TDA_0.5_ catalyst. The formation of n-p type homojunction and decrement in the charge transfer resistance is highly desirable for high photocatalytic performance.

### Photocatalytic dye degradation experiments

**Figure 5:**
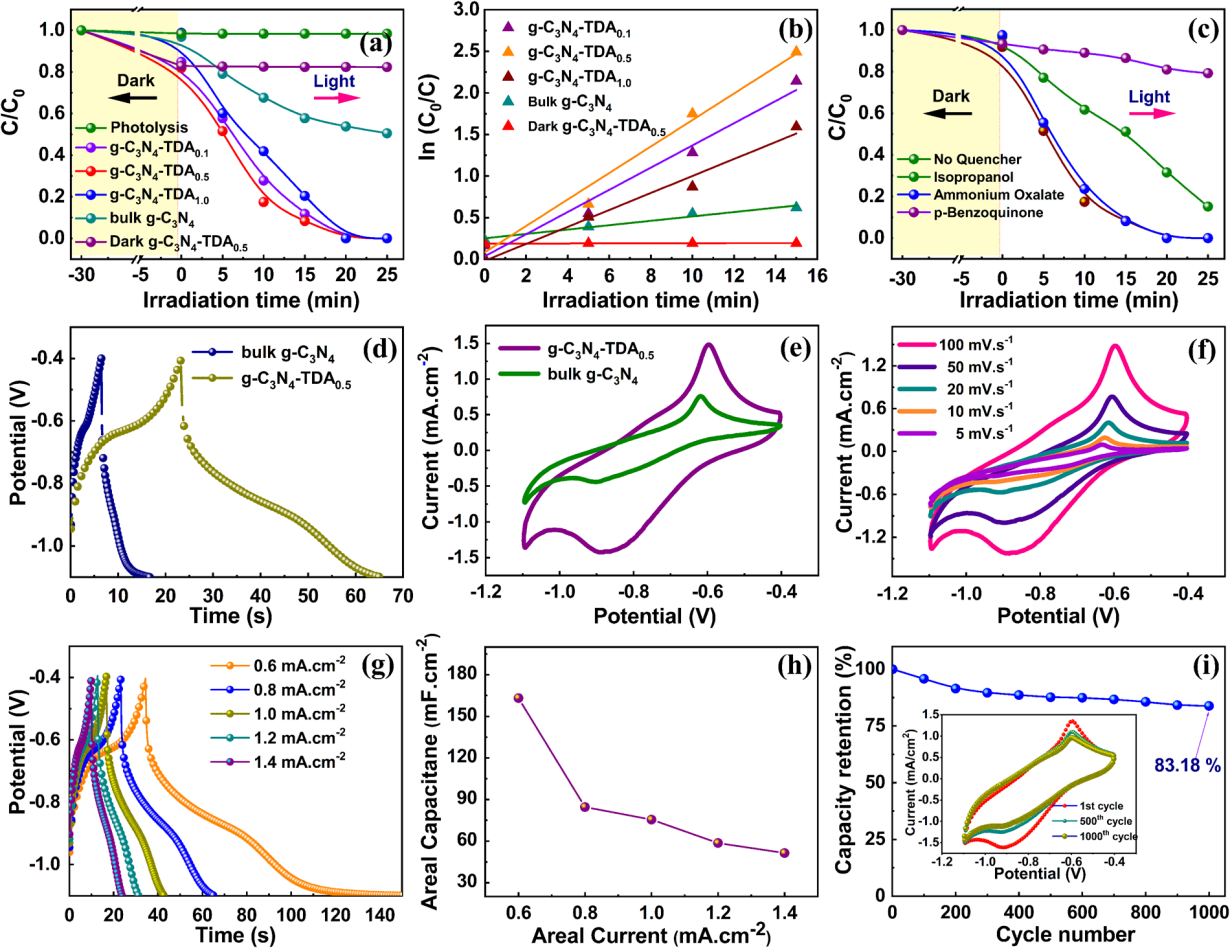
(**a**) Photocatalytic AV 7 (20 mg/L) degradation performance (**b**) reaction kinetics of AV 7 degradation (**c**) reactive species trapping experiments over g-C_3_N_4_-TDA_0.5_ photocatalyst (**d**) comparative cyclic voltammograms in KOH (1.0 M) solution measured at scan rate of 100 mV/s, and (**e**) comparative galvanostatic charge-discharge profiles at current load of 0.8 mA/cm^−2^ using SS electrode coated with g-C_3_N_4_ and g-C_3_N_4_-TDA_0.5_ samples (**f**) cyclic voltammograms of g-C_3_N_4_-TDA_0.5_ at different scan rates (5–100 mV.s^−1^) (**g**) charge-discharge curves of g-C_3_N_4_-TDA_0.5_ electrode at different current density profiles (0.6–1.4 mA.cm^−2^) (**h**) areal capacitance of g-C_3_N_4_-TDA_0.5_ at different areal currents (**i**) cyclic stability of g-C_3_N_4_-TDA_0.5_ electrode at scan rate of 100 mV.s^−1^ for 1000 cycles. The inset of (**i**) shows cyclic voltammograms for 1^st^, 500^th^ and 1000^th^ cycle.

The photocatalytic performance of grafted g-C_3_N_4_ is tested for the degradation of an aqueous solution of AV7 dye (20 mg/L) under visible light irradiation. The grafted g-C_3_N_4_-TDA samples have shown rapid photodegradation performance than bulk g-C_3_N_4_ **(**Fig. 5a**)**. Moreover, g-C_3_N_4_-TDA_0.5_ sample displayed a high rate kinetics in AV 7 degradation under 15 min of visible light irradiation **(**Fig. 5b**)**. The rate constant (*k*) found for the g-C_3_N_4_-TDA_0.5_ sample (*k* = 0.1591 min^−1^) was virtually 5-fold higher than bulk g-C_3_N_4_ (*k* = 0.0381 min^−1^). The grafting of TDA molecule can extend the visible light adsorption, lowers charge carrier recombination and improves the surface area which could be the reasons for the high photocatalytic performance of g-C_3_N_4_-TDA samples. The photocatalytic control ability of bulk g-C_3_N_4_ and g-C_3_N_4_-TDA_0.5_ under dark were also evaluated and has shown negligible degradation kinetics. The photolysis of AV 7 under visible light irradiation can be ruled out as a blank experiment under light without photocatalyst could not promote degradation of AV 7.

For unambiguous identification of reactive oxygen species (ROS) involved in the dye degradation process, carrier trapping experiments were performed with scavengers such as isopropanol (2 mL) for hydroxyl radicals (${{\rm{OH}}}^{\bullet }$), *p*-benzoquinone (10^−3^ M, 5 mL) for superoxide (${{\rm{O}}}_{2}^{\bullet -}$) and ammonium oxalate (0.1 g) for photon $({{\rm{h}}}^{+})$ quenching. Interestingly, only *p*-benzoquinone found to effectively suppress the degradation rate, which indicates the role of ${{\rm{O}}}_{2}^{\bullet -}$ as major reactive species in the system **(**Fig. 5c**)**. The photogenerated ${{\rm{O}}}_{2}^{\bullet -}$ can produce highly reactive ${{\rm{OH}}}^{\bullet }$ which further improves the degradation process as evidenced by carrier trapping experiments.

Predicated on carrier trapping experiments a possible mechanism for degradation of AV 7 is proposed as follows.

1 $${\rm{g}} \mbox{-} {{\rm{C}}}_{3}{{\rm{N}}}_{4}{ \mbox{-} \mathrm{TDA}}_{{\rm{0.5}}}+{\rm{h}}\nu \to {{{\rm{h}}}^{+}}_{(\mathrm{VB})}+{{{\rm{e}}}^{-}}_{(\mathrm{CB},\mathrm{TDA})}$$

2 $${{\rm{O}}}_{2}+{{{\rm{e}}}^{-}}_{({\rm{C}}{\rm{B}},{\rm{g}}-{{\rm{C}}}_{3}{{\rm{N}}}_{4}-{\rm{T}}{\rm{D}}{{\rm{A}}}_{0.5})}\to {{{\rm{O}}}_{2}}^{-\bullet }(\,+\,0.2\,{\rm{V}})$$

3 $${{{\rm{O}}}_{2}}^{-\bullet }+2{{\rm{H}}}_{2}{\rm{O}}+2{{{\rm{e}}}^{-}}_{({\rm{C}}{\rm{B}})\to 3{{\rm{O}}{\rm{H}}}^{-}}+{{\rm{O}}{\rm{H}}}^{\bullet }(\,+\,0.9\,{\rm{V}})$$

4 $$\begin{array}{c}{{\rm{OH}}}^{-}+{{{\rm{h}}}^{+}}_{(\mathrm{VB})}\to {{\rm{OH}}}^{\bullet }(\,+\,{\rm{1.3}}\,{\rm{V}})\end{array}$$

$$\begin{array}{c}{\rm{AV}}\,{\rm{7}}+{{\rm{OH}}}^{\bullet }\to {\rm{degradation}}\,{\rm{product}}\\ {\rm{AV}}\,{\rm{7}}+{{{\rm{h}}}^{+}}_{(\mathrm{VB})}\to {\rm{degradation}}\,{\rm{products}}\end{array}$$

The visible light irradiation of g-C_3_N_4_-TDA_0.5_ photocatalyst could excite valence band (VB) electrons of g-C_3_N_4_. These excited electrons further may react with surface bound oxygen to generate ${{\rm{O}}}_{2}^{\bullet -}$, and ${{\rm{O}}}_{2}^{\bullet -}$ later combine with water to form ${{\rm{OH}}}^{\bullet }.$ As the VB of g-C_3_N_4_ is less positive than the standard reduction potential of ${{\rm{OH}}}^{\bullet }\,(2.68\,{\rm{eV}}\,{\rm{vs}}\,{\rm{SHE}}),$ VB ${{\rm{h}}}^{+}$ could not oxidise H_2_O into ${{\rm{OH}}}^{\bullet }$ thus reaction given in Eq. 4 is not feasible in the present system but VB ${{\rm{h}}}^{+}$ directly involved in the degradation of AV 7. The synergistic effect of ${{\rm{O}}}_{2}^{\bullet -}$ and ${{\rm{h}}}^{+}$ contribute to the rapid degradation of AV 7.

From the reproducibility perspective, the degradation experiments were performed for successive repeated cycles of the same catalyst. It can be found that g-C_3_N_4_-TDA_0.5_ photocatalyst is stable and has shown a small decrease in photoactivity after each repeated cycle. This could be due to loss of catalyst during repeated washings and centrifugation or the presence of adsorbed dye residues on the active sites of the catalyst. Therefore, to regenerate the active sites on the surface of the catalyst, recycled photocatalyst heated at 150 °C for 2 h, has exhibited almost similar photoactivity as of fresh sample (Fig. S3). The FE-SEM image of the recycled g-C_3_N_4_-TDA_0.5_ catalyst has shown identical morphological features as of pristine catalyst **(**Fig. S4**)**, suggesting the robustness of the catalyst. These results demonstrate the long term durability of the metal-free catalyst for practical applications.

### Electrochemical capacitance behaviour of g-C_3_N_4_-TDA_0.5_

To understand the electrochemical capacitive behaviour of g-C_3_N_4_-TDA_0.5_, cyclic voltammetry (CV) and galvanostatic charge-discharge (GCD) techniques were employed. In the comparative charge-discharge approach, g-C_3_N_4_-TDA_0.5_ profile exhibited a non-ideal discharge curve with higher discharging time than bare g-C_3_N_4_ **(**Fig. 5d**)**. The comparative CV analysis of bare g-C_3_N_4_ and g-C_3_N_4_-TDA_0.5_ at a fixed scan rate of 100 mV.s^−1^ displayed a regular rectangular-shape with a pair of redox peaks, which indicates the pseudocapacitive character of g-C_3_N_4_ based materials **(**Fig. 5e**)**. The formation of redox peaks in the CV curve corresponds to the redox reaction between pyridinic nitrogen of carbon nitride and electrolyte^56^. The CV of g-C_3_N_4_-TDA displayed a large capacitive area than bulk g-C_3_N_4_, suggesting the improved ion transport and storage capacitance in the g-C_3_N_4_-TDA_0.5_. This could be due to extended π-conjugation in g-C_3_N_4_ by the addition of electron acceptor TDA molecule.

Further to evaluate the enhanced capacitive behaviour of g-C_3_N_4_-TDA_0.5_, the CV measurements were performed at different scan rate ranging from 5 to 100 mV.s^−1^_._ The CV curves showed a gradual increase in corresponding currents, which implies the capacitive behaviour of g-C_3_N_4_-TDA_0.5_ electrode **(**Fig. 5f**)**. Almost a regular rectangle is preserved even at a higher scan rate of 100 mV.s^−1^, which reflects the enhanced capacitive behaviour, high rate capability and the effective ion transport in the g-C_3_N_4_-TDA_0.5_ electrode. This enhanced capacitive performance is may be due to the modified electronic structure of g-C_3_N_4_. Furthermore, the galvanostatic charge-discharge (GCD) approach was conducted at different current densities in 1 M KOH electrolyte **(**Fig. 5g**)**. The GCD profiles at different current loads reflect short charging time and a longer discharging time with initial voltage drop, which indicates the pseudocapacitive behaviour of the g-C_3_N_4_-TDA_0.5_ electrode^57^. The areal capacitance of electrode material was calculated from the integrated area of corresponding charge/discharge curves using the earlier reported equation^58^. The estimated areal capacitance of the g-C_3_N_4_-TDA_0.5_ at 0.5, 0.8, 1.0, 1.2, and 1.4 mA.cm^−2^ current loads were 163.17, 84.58, 75.51, 58.62 and 51.42 mF.cm^−2^ respectively **(**Fig. 5h**)**. To our delight, the g-C_3_N_4_-TDA_0.5_ electrode has an about 6-fold increase in areal capacitance than bare g-C_3_N_4_ material. The better electrochemical performance of g-C_3_N_4_-TDA_0.5_ is may be due to the high surface area which could provide a large contact surface for electrode and electrolyte, nitrogen richness and the addition of aromatic thiophene could also enhance the easy mass diffusion. In addition, we also tested the recycling ability of g-C_3_N_4_-TDA_0.5_ electrode at a fixed scan rate of 100 mV.s^−1^, which has shown capacity retention of 83% even after 1000 repeated cycles **(**Fig. 5i**)**. This result, indicate the noticeable capacity retention and long term durability of electrode material for high-performance pseudocapacitors.

5 $${\rm{g}} \mbox{-} {{\rm{C}}}_{3}{{\rm{N}}}_{4}.{\rm{TDA}}+{{\rm{xH}}}^{+}+{{\rm{yK}}}^{+}+{{\rm{xye}}}^{-}\leftrightarrow {\mathrm{Hxg} \mbox{-} {\rm{C}}}_{3}{{\rm{N}}}_{4}.{\rm{Ky}}.{\rm{TDA}}$$

The mechanism involved for charge storage in the g-C_3_N_4_-TDA_0.5_ electrode is attributed to the intercalation/de-intercalation of cations on the surface of the electrode by means of rapid redox reactions as in Eq. 5. Moreover, the two-dimensional layered surface morphology of g-C_3_N_4_-TDA_0.5_ is useful to enhance the effective utilisation of active electrode material in intercalation/deintercalation of electrolyte ions^59^. These obtained results pave incipient approach in the fabrication of potential g-C_3_N_4_ based supercapacitors with anchored organic moieties for large surface area, a high degree of nitrogen and extended aromatic functional groups for rapid and easy charge transport.

In summary, a facile post modification strategy was used to graft organic TDA moiety at defect sites of g-C_3_N_4_. Addition of electron acceptor TDA molecule at terminal amino groups of n-type g-C_3_N_4_ resulted in the formation of n-p type homostructural g-C_3_N_4_ photocatalyst. The formation of n-p type homojunction induce built in interfacial charge separation and enable opposite migration of electron-hole pairs that minimises recombination phenomena. As obtained n-p type g-C_3_N_4_ homojunction has a significant enhancement in photo-degradation performance of AV 7 dye and high charge-capacitance behaviour. The current results are captivating in designing of n-p type g-C_3_N_4_ homojunctions using various acceptor molecules. The basic idea is to choose a simple organic acceptor molecule that is capable of binding at –NH_2_ sites of g-C_3_N_4,_ of transferring electrons in one component and holes in the reverse direction within in the same homojunction. The insights in the modification of g-C_3_N_4_ surface functional groups can have more importance in the development of high-performance g-C_3_N_4_ based photocatalytic materials. We believe that our results pave new avenues in the building of novel class of n-p type homostructural g-C_3_N_4_ based photocatalysts.

## Materials and Methods

All the chemicals, melamine (Finar Chem. Ltd, 99%), 2,5-thiophene dicarboxylic acid (Aldrich, 99%), Acid Violet 7 (Aldrich, dye content ca. 85%), were of analytical grade and used without any further purification. Millipore water (conductivity <0.15 mS cm^−1^) was utilized throughout the experiments.

### Synthesis of bulk g-C_3_N_4_

Bulk carbon nitride (g-C_3_N_4_) was synthesized according to the earlier reported literature^48^, 5 g of melamine was heated in a caped alumina crucible at 550 °C for 2 h under air with a heating rate of 10 °C/min, a yellow product obtained is labelled as bulk g-C_3_N_4_.

### Grafting of g-C_3_N_4_ with 2, 5-dicarboxylic thiophene

A series of 2, 5-thiophene dicarboxylic acid (TDA) grafted g-C_3_N_4_ samples were prepared by mixing bulk g-C_3_N_4_ (1 g) with TDA (0.1, 0.5 and 1 mM) and thoroughly ground for 30 min using mortar and pestle. The corresponding mixture (g-C_3_N_4_-TDA_*x*_) was transferred into a beaker containing 50 ml methanol, capped and placed on a heating mantle with continuous stirring at 90 °C for the complete evaporation of a solvent. After cooling to room temperature, as the obtained yellow product is labelled as TDA grafted carbon nitride (g-C_3_N_4_-TDA_*x*_), where *x* = 0.1, 0.5 and 1 mM of TDA per gram of g-C_3_N_4_.

### Photocatalytic study

Photocatalytic activity of the prepared catalysts was evaluated by degradation of an aqueous solution of AV 7 dye (20 mg/L) under sunlight type irradiation in homemade photo-irradiator^60^. Briefly, for each experiment 100 mL of dye solution (20 mg/L) was taken in a beaker with glass lidand0.1 g of catalyst was suspended in it. Before light irradiation, catalysts were immersed in AV 7 dye solution for 30 min to attain the adsorption-desorption equilibrium at ambient conditions. Magnetic stirring was performed throughout the experiment for the homogenization of suspension. At regular time intervals, an aliquot was withdrawn, centrifuged and measured the corresponding absorbance at λ max = 523 nm using UV-visible spectrophotometer (Shimadzu1800). Photolysis reaction was checked by irradiating AV 7 dye solution without catalyst, and the concentration was monitored periodically.

### Materials characterisation

XRD analysis of the samples was performed in the 2θ range of 10° and 80° using an X-ray diffractometer (Rigaku: Miniflex-II-DD34863) with Cu Kα radiation (λ = 0.15418 nm) operated at 30 kV and 15 mA at a scan rate of 5° min^−1^. X-ray photoelectron spectroscopy (XPS) studies were carried out on a VG Microtech electron spectrometer using Mg Kα X-rays (hν = 1253.6 eV) as the primary source of radiation. The chamber pressure was maintained at 1 × 10^−9^ Torr. Appropriate correction for charging effect was made with the help of the C 1 s signal appearing at 284.5 eV. XPS Peak 4.1 software was used to fit the XPS peaks. The decomposition of the spectra curves into individual components was performed using a combination of Gaussian and Lorentzian functions after background subtraction by the Shirley method. The TEM images were taken using a FEI Tecnai T-20 electron microscope operating at 300 kV. Energy dispersive X-ray spectroscopy analysis was carried out using Zeiss FESEM. Solid state ^13^C nuclear magnetic resonance (NMR) patterns was recorded using a Bruker Avance III 400 MHz NMR machine with basic frequency of 104.22 MHz. Single pulse experiment with pulse duration of 6 ms with a relaxation delay time of 8 s was used for recording the spectra. The samples were packed in 4 mm zirconia rotors and subjected to a spinning speed of 5 kHz. NMR Chemical shifts are reported with respect to TMS as an external reference.UV-visible diffuse reflectance spectra (UV-Vis DRS) of all samples were recorded using a Jasco (model V-670) spectrophotometer equipped with an integrating sphere accessory. Barium sulfate was used as reference for the reflectance spectra.

### Photoelectrochemical study

Electrochemical studies were carried out by a PARSTAT 4000 (Princeton Applied Research, Ametek, USA) Potentiostat/Galvanostat using a conventional three-electrode system. The electrochemical characterization of prepared samples was measured by coating as prepared materials on stainless steel (SS) substrate (1 cm × 5 cm, 305 grades). The cyclic voltammetry (CV) analysis and galvanostatic charge-discharge (GCD) techniques were performed by immersing coated SS electrode in a saturated aqueous solution of 1 M KOH.

## Supplementary information

Extended π-conjugative n-p type homostructural graphitic carbon nitride for photodegradation and charge-storage applications
