# Supplementary material for: Extended π-conjugative n-p type homostructural graphitic carbon nitride for photodegradation and charge-storage applications
Source: Sci Rep. 2019 May 10;9:7186. doi: 10.1038/s41598-019-43312-5 (PMC6510722; doi:10.1038/s41598-019-43312-5)
Supplement: Supplementary file 1 — Extended π-conjugative n-p type homostructural graphitic carbon nitride for photodegradation and charge-storage applications [file 41598_2019_43312_MOESM1_ESM.docx]

Supporting Information

Extended π-conjugative n-p type homostructural graphitic carbon nitride for photodegradation and charge-storage applications

Devthade Vidyasagar^†a^, Sachin G. Ghugal^†b^, Suresh S. Umare*^a^, Murali Banavoth*^b^

*^a^Materials and Catalysis Laboratory, Department of Chemistry, Visvesvaraya National Institute of Technology (VNIT), Nagpur, 400010 India*

*^b^Solar Cells and Photonics Research laboratory, School of Chemistry, University of Hyderabad, Hyderabad, Telangana, 500046 India*

Corresponding Authors:

**E-mail:** [ssumare@chm.vnit.ac.in](mailto:ssumare@chm.vnit.ac.in), **Fax:** +91 712 2223230, **Tel:** +91 712 2801316

**E-mail:** [murali.banavoth@uohyd.ac.in](mailto:murali.banavoth@uohyd.ac.in), **Fax:** +91 40 2313 8400, **Tel:** +91 40 2313 8428


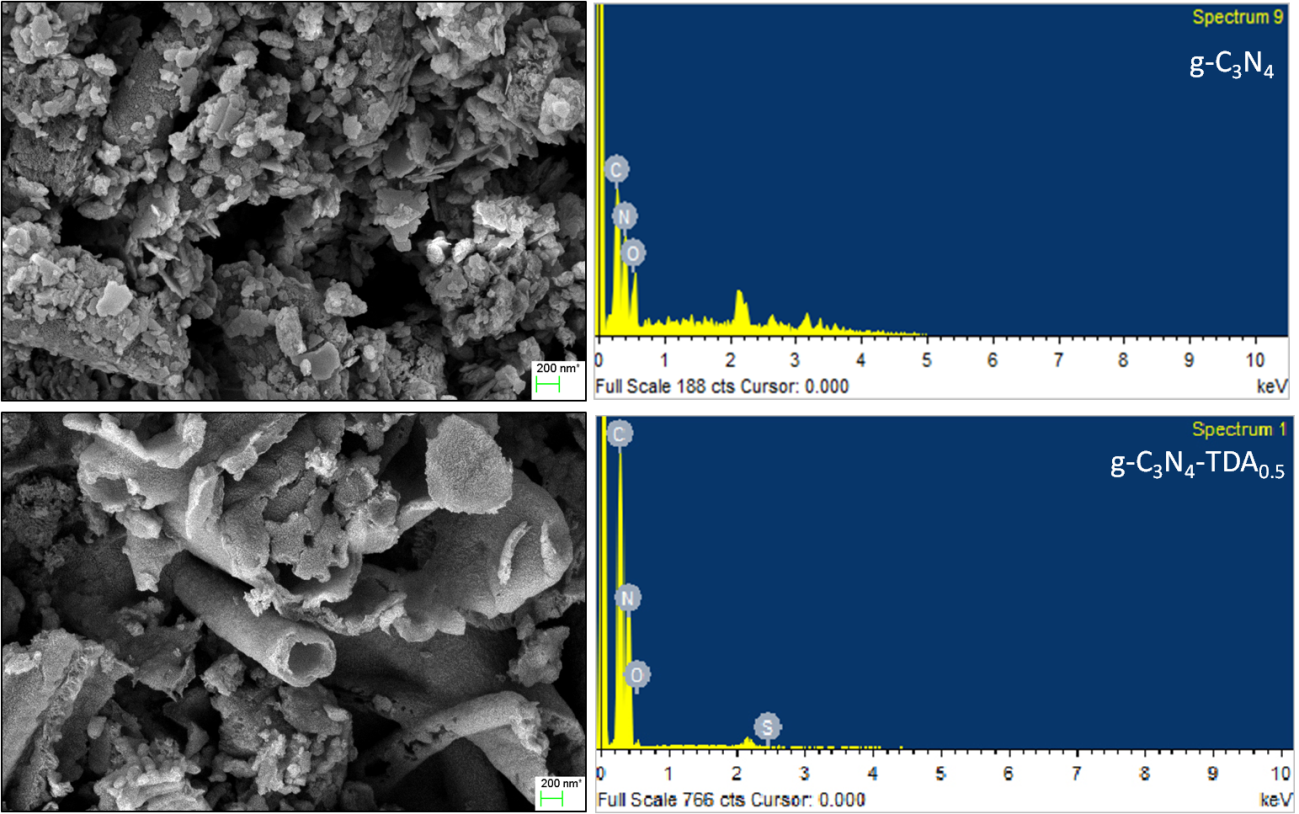


b

d

c

a

**Figure S1**. FE-SEM image and EDS mapping of g-C_3_N_4_ (a, b) and g-C_3_N_4_-TDA_0.5_ (c, d).


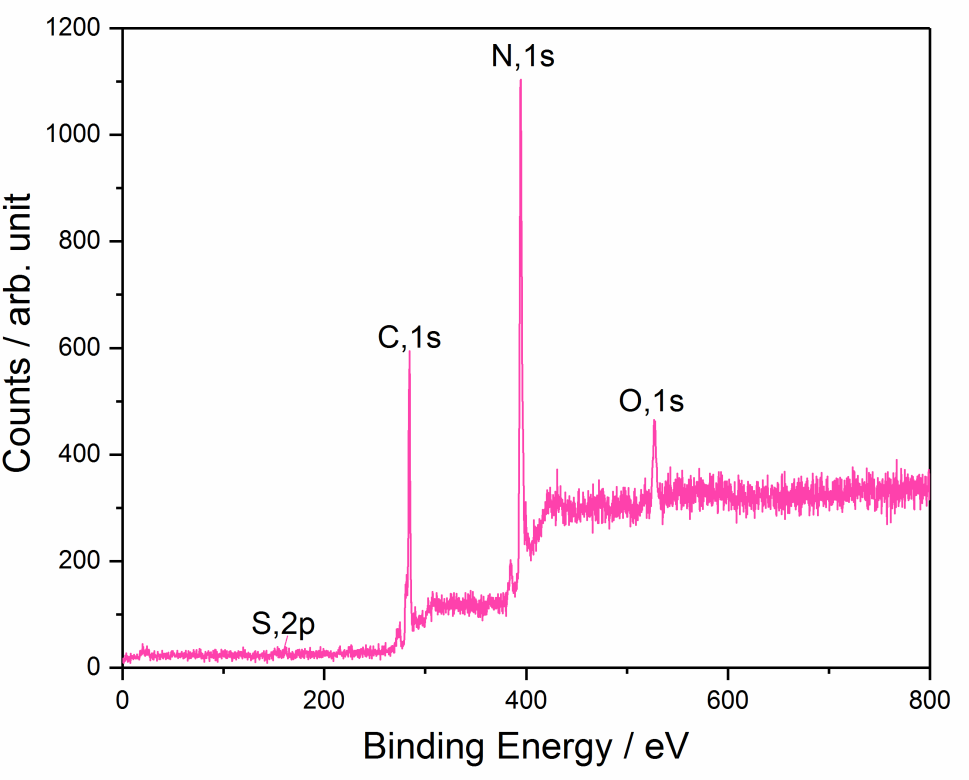


**Figure S2.** XPS survey spectrum of g-C_3_N_4_-TDA_0.5_.





**Figure S3.** Photodegradation performance of g-C_3_N_4_-TDA_0.5_ sample for consecutive five repeated cycles under visible-light irradiation.

**
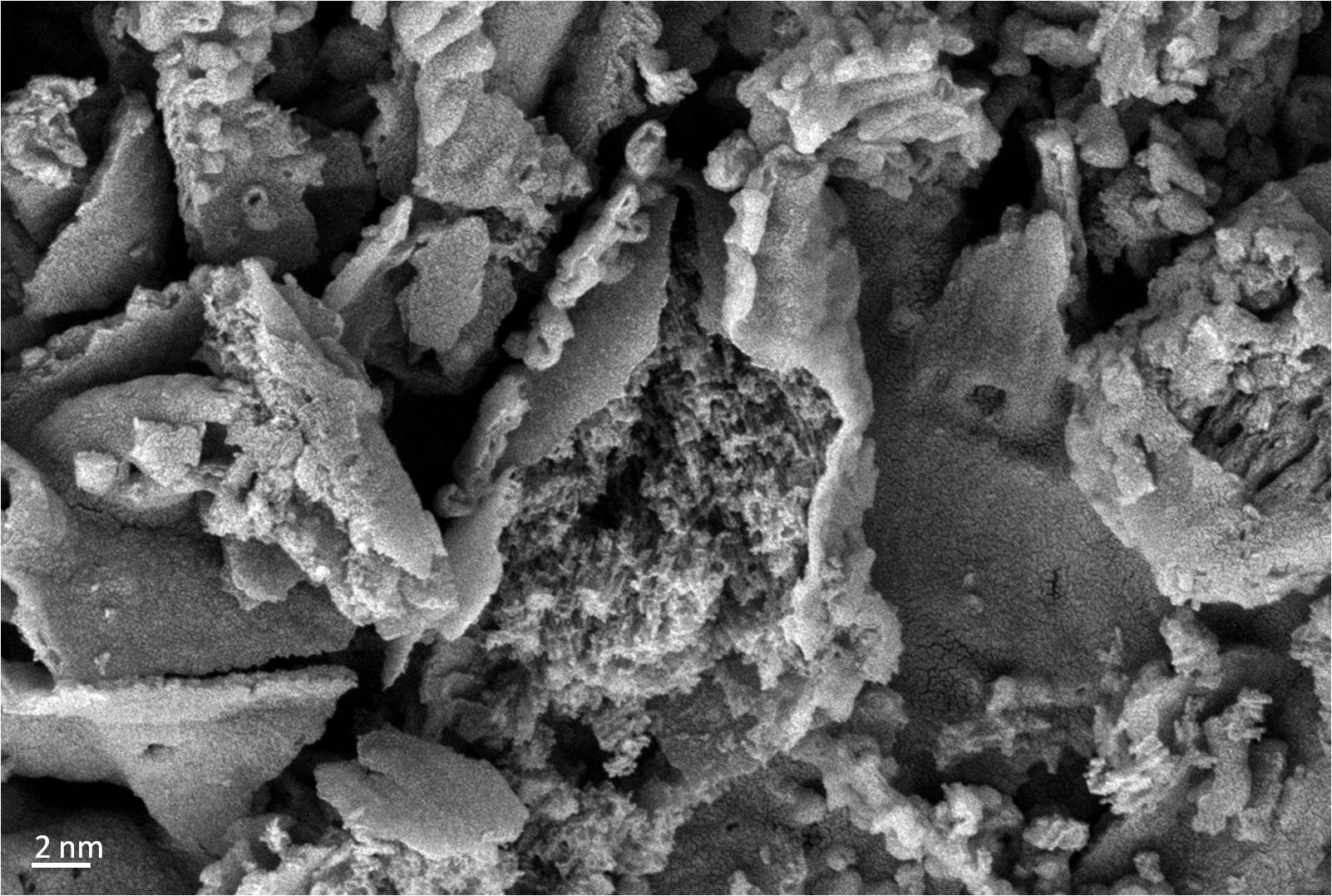
**

**Figure S4.** FE-SEM image of g-C_3_N_4_-TDA_0.5_ catalyst after four repetitive cycles.
